# Dynamics of host immune response development during *Schistosoma mansoni* infection

**DOI:** 10.3389/fimmu.2022.906338

**Published:** 2022-07-08

**Authors:** Alice H. Costain, Alexander T. Phythian-Adams, Stefano A. P. Colombo, Angela K. Marley, Christian Owusu, Peter C. Cook, Sheila L. Brown, Lauren M. Webb, Rachel J. Lundie, Jessica G. Borger, Hermelijn H. Smits, Matthew Berriman, Andrew S. MacDonald

**Affiliations:** ^1^ Lydia Becker Institute of Immunology and Inflammation, University of Manchester, Manchester, United Kingdom; ^2^ Department of Parasitology, Leiden University Medical Center, Leiden, Netherlands; ^3^ Wellcome Sanger Institute, Wellcome Genome Campus, Hinxton, United Kingdom; ^4^ Medical Research Council Centre for Medical Mycology, University of Exeter, Exeter, United Kingdom; ^5^ Department of Immunology, University of Washington, Seattle, WA, United States; ^6^ 360biolabs, Melbourne, VIC, Australia; ^7^ The Walter and Eliza Hall Institute, VIC, Australia; ^8^ Wellcome Centre for Integrative Parasitology, University of Glasgow, Glasgow, United Kingdom

**Keywords:** schistosomiasis, dendritic cells, pathology, chronic infection, transcriptomic (RNA-seq)

## Abstract

Schistosomiasis is a disease of global significance, with severity and pathology directly related to how the host responds to infection. The immunological narrative of schistosomiasis has been constructed through decades of study, with researchers often focussing on isolated time points, cell types and tissue sites of interest. However, the field currently lacks a comprehensive and up-to-date understanding of the immune trajectory of schistosomiasis over infection and across multiple tissue sites. We have defined schistosome-elicited immune responses at several distinct stages of the parasite lifecycle, in three tissue sites affected by infection: the liver, spleen, and mesenteric lymph nodes. Additionally, by performing RNA-seq on the livers of schistosome infected mice, we have generated novel transcriptomic insight into the development of schistosome-associated liver pathology and fibrosis across the breadth of infection. Through depletion of CD11c^+^ cells during peak stages of schistosome-driven inflammation, we have revealed a critical role for CD11c^+^ cells in the co-ordination and regulation of Th2 inflammation during infection. Our data provide an updated and high-resolution account of how host immune responses evolve over the course of murine schistosomiasis, underscoring the significance of CD11c^+^ cells in dictating host immunopathology against this important helminth infection.

## Introduction

Schistosomiasis is a complex and potentially life-threatening parasitic disease, caused by infection with blood flukes of the genus *Schistosoma. S. mansoni*, S. *haematobium* and S. *japonicum* are responsible for most human cases, with approximately 240 million individuals infected globally, predominantly clustering in specific regions of Africa, Asia, the Middle East, and South America (1).

Schistosomiasis has a distinctive triphasic immune profile that reflects transformative events within the parasite lifecycle (2). In brief, the host first progresses through a (pre-patent) acute phase (0-5 WPI; wks post infection), where it responds to immature, lung migrating schistosomula and developing worm pairs (3). In the second (post-patent) acute phase (5-12 WPI), the host is confronted daily with hundreds of highly immunogenic eggs, and must contend with accompanying inflammation and associated tissue damage (4). In the final chronic phase (12 WPI+), the host enters a more regulated or ‘exhausted’ state that follows prolonged exposure to parasite antigens (Ags) (5, 6). At each lifecycle stage schistosomes employ a wide range of immune evasion and manipulation strategies that enable mitigation against collateral tissue damage and promotion of long-term survival within the host (7). For example, as an appropriately regulated T Helper (Th) 2 response is essential for host and parasite survival (8–10), schistosomes produce an array of molecules that guide host immunity towards a Type 2 phenotype (11–17), whilst concurrently encouraging the expansion of regulatory networks that downregulate and resolve chronic inflammation (14, 15, 18–20).

The immune response triggered at each lifecycle stage has been thoroughly dissected in experimental murine infections and, to a lesser extent, human and primate work. Together, the following immune narrative has been established: during the initial 4-5 wks of pre-patency, migrating schistosomula and juvenile worms evoke a low-level mixed T helper 1 (Th1)/Th2 immune response (21–23). This profile takes a dramatic shift at around 5-6 WPI, with egg production skewing host immunity towards a Th2-dominated profile, including dramatic upregulation of interleukin (IL-) 4, IL-5, IL-13, IgE and eosinophilia. This Type 2 environment dwarfs and counteracts the early Type 1 component (21), peaks approximately 8-10 WPI, and is critical for coordinated granulomatous inflammation (8, 10, 24). Moreover, although Th17 responses are acknowledged during schistosomiasis, they are generally considered low level in relation to Th2 or Th1 responses, and typically only emerge in specific mouse strains (i.e. CBA mice but not C57BL/6 or BALB/C) (25, 26). The majority of data suggests Th17 responses act to promote immune pathology rather than benefit the host (27, 28). As infections progress into chronicity, Type 2 responses decline and regulatory responses prevail (5, 29–31). Down-modulation of Type 2 responses and suppression of severe disease is thought to be primarily mediated by IL-10 (32), with its secretion attributed to Regulatory B cells (Bregs) (33, 34) and T cells (Tregs) (30, 35–37). Tissue fibrosis (scarring) is central to schistosomiasis associated pathology, particularly in the liver, with IL-13 proposed as the main pro-fibrotic mediator in mice (38). This immunological picture has been established over decades of research, often focusing on a single tissue site, timepoint or event within the schistosome lifecycle. While time-course studies exist, very few have simultaneously evaluated the Th1/Th2/regulatory balance over the course of infection, in a range of tissue sites. Pioneering work in the early 1990s provided two splenic time-course studies, which form the basis of our understanding of the Th1-Th2 switch from the point of egg production (21, 22). However, these studies reflect the technical limitations of the time, and have received minimal follow-up with the resolution that is possible today.

Dendritic cells (DCs) are central players in the host immune system, poised to recognize and respond to invading threats and inflammation, and translate these danger signals into the activation and differentiation of T cells (39). DCs have proven key to orchestration of Th1 and Th2 inflammation, as well as the promotion of regulatory responses. In the context of schistosomiasis, we have previously shown that they play a fundamental role in the priming of CD4^+^ T cell responses, with depletion of CD11c^+^ DCs early in murine *S. mansoni* infection (wks 4 – 6) leading to a stark reduction in splenic and hepatic Th2 cells (IL-4, IL-5, and IL-13) and Tregs (24). However, the involvement of DCs in maintenance and/or regulation of schistosome infection beyond wk 6 remains unclear. Similarly, it is currently unknown whether DCs in later stages of infection remain vital for coordinating CD4^+^ T cell function and cytokine secretion, or if other antigen presenting cells (APCs) adopt more dominant roles in this context.

We now provide a comprehensive account of schistosome-elicited immune responses over the course of infection and across multiple tissue sites (liver, spleen, and mesenteric lymph nodes [MLNs]). In addition to refined characterization of cell populations and cytokine dynamics across these compartments, we have interrogated how the host responds to infection and attempts to resolve egg-driven damage by RNA-seq analysis of liver samples isolated over the course of infection. Finally, we have revealed a critical role for CD11c^+^ cells in the maintenance of hepatic Type 2 inflammation, with CD11c depletion from 6 WPI leading to compromised cytokine production and altered granulomatous pathology. These novel data elevate and update our fundamental understanding of immune response development over the course of murine schistosomiasis, and the significance of CD11c^+^ cells in coordinating immunopathology against this important and relevant helminth infection.

## Materials and Methods

### Mice

Experiments were performed using female C57BL/6 mice, CD11c.DOG (40) x C57BL/6 or CD11c.DOG x 4get IL-4-eGFP F1 mice (41), housed under specific pathogen free conditions at the University of Manchester or the University of Edinburgh, and used at 8-12 wks of age. Experiments were performed under a project license granted by the Home Office UK following ethical review by the University of Manchester or University of Edinburgh, and performed in accordance with the United Kingdom Animals (Scientific Procedures) Act of 1986.

### *Schistosoma mansoni* Infections and Cell Depletions

*Biomphalaria glabrata* snails infected with *S. mansoni* parasites were obtained from Biomedical Research Institute, Rockville, MD. Mice were percutaneously infected (42) with 40-80 *S. mansoni* cercariae, with infections lasting 4, 6, 8, 12 or 15 wks in duration. To quantify parasite burden, *S. mansoni* eggs were isolated and counted from pieces of liver (approx. 1g in weight) or intestine, and digested overnight in 5% KOH (42). For CD11c depletion experiments, mice received daily intraperitoneal injections with 8 ng/g diphtheria toxin (Sigma-Aldrich) in PBS or with PBS alone (24, 40), from wk 6 of infection (days 42-51), and culled on day 52.

### Cell Isolation

Single-cell suspensions of liver, MLN and spleen were prepared using the following methods, and as described previously (24). Spleens and MLNs were diced and digested at 37°C with 0.8 U/ml Liberase TL and 80 U/ml DNase I type IV in HBSS (all Sigma), supplemented with 50 U/ml penicillin and 50 µg/ml streptomycin (Invitrogen). After 30 min the digestion reaction was halted by the addition of 100 µl/ml 0.1 M, pH 7.3, EDTA (Ambion) stop solution, followed by addition of DMEM containing 50 U/ml penicillin and 50 µg/ml streptomycin. This was then passed through a 70 µm cell strainer to generate a cellular suspension, with splenocytes undergoing an additional RBC lysis incubation to remove contaminating erythrocytes. Livers were perfused, minced with sterile scalpel blades, and incubated at 37°C for 45 min using the digestion media detailed above. After addition of EDTA stop solution and DMEM, digested livers were passed through a 100 µm cell strainer. To separate leukocytes, liver cells were resuspended in 33% isotonic Percoll (GE healthcare) and centrifuged at 700 g. Pelleted cells were passed through a 40 µm cell strainer (for removal of *S. mansoni* eggs), followed by RBC lysis, counting and resuspension in PBS supplemented with 2% FBS, 2 mM EDTA, for flow cytometry or culture/stimulation.

### Quantification of Cytokines

Cytokine assays were performed as previously described (43, 44). Single-cell suspensions of livers, MLNs and spleens were stimulated *ex vivo* with plate bound anti-CD3 (0.5 μg/well) or SEA (0.25 µg/well) in X-vivo 15 media (Lonza) supplemented with 2 mM l-Glutamine and 50 µM 2-ME (Sigma). Stimulation was carried out in 96 well U bottom plates, with 1 x 10^6^ cells seeded per well in 200µl volumes. Cell supernatants were harvested 72 h later and stored at -20°C until further analysis. Paired capture and detection Abs were used for analysis of murine IL-4, IL-10, IL-17A (Biolegend) IL-5, IFNγ (produced from hybridomas in-house) and IL-13 (eBioscience).

### Serum Antibody and IgE Levels

SEA-directed antibodies and IgE levels were measured as described previously (24). In brief, to quantify serum levels of SEA-specific IgG, IgG1, IgG2a, IgG2c and IgG3, 96 well plates (NUNC Maxisorp) were coated with 5 μg/mL SEA overnight, then blocked with 1% bovine serum albumin (BSA, Sigma) in PBS for 1 h at room temperature. Plates were then incubated with sera at doubling dilutions (1:50-1:6400). Alkaline phosphatase conjugated goat anti mouse IgG, IgG1, IgG2a, IgG2c and IgG3 (SouthernBiotech) were added to plates, followed by liquid PNPP substrate i.e. ‘liquid PNPP substrate (Thermo Scientific), with absorbances read at 405 nm. Serum levels of IgE (BD) were measured using paired capture and biotinylated detection antibodies, with quantity assessed *via* standard curve and absorbances read at 450 nm. For all ELISAs, between individual incubation steps, plates were washed 3 times with PBS containing 0.05% Tween (Sigma). Data presented as endpoint dilutions.

### Flow Cytometry

Single cell suspensions were washed with PBS and stained for viability with ZombieUV (1:2000; Biolegend) or LIVE/DEAD™ Fixable Aqua (1:500; Invitrogen). Cells were then incubated with 5 μg/ml FC block (αCD16/CD32; 2.4G2; Biolegend) in FACS buffer (PBS containing 2% FBS and 2 mM EDTA), before staining for surface markers for 30 min. After surface staining, cells were washed twice with FACS buffer before fixation in 1% PFA for 10 min. For intracellular staining, cells were permeabilized with eBio Fixation/Permeabilisation buffer for 1 h, before staining with relevant intracellular antibodies for 30 min. Samples were analyzed by flow cytometry (LSR Fortessa, BD) and data analyzed using FlowJo v10 software. A list of antibodies used is provided in **Table 1**.

**Table 1:** List of flow cytometry antibodies and their clones.

Company	Target	Clone
BD	Siglec-F	E50-2440
Biolegend	CD11b	M1/70
Biolegend	CD11c	N418
Biolegend	CD169	3D6.112
Biolegend	CD172a	P84
Biolegend	CD26	DPP-4
Biolegend	CD4	RM4-5
Biolegend	CD45	30-f11
Biolegend	CD64	x54-5/7.1
Biolegend	CD8	53-6.7
Biolegend	F4/80	BM8
Biolegend	IFNy	XMG1.2
Biolegend	IL-10	JES5-16E3
Biolegend	IL-17	TC11-1810.1
Biolegend	IL-4	11B11
Biolegend	Ly6C	HK1.4
Biolegend	Ly6G	1A8
Biolegend	PDCA-1	927
Biolegend	XCR1	ZET
Ebioscience	B220	RA-6B2
Ebioscience	IL-13	ebio13A
Ebioscience	IL-5	TRFK.5
Ebioscience	MHCII	MS/114.15.2
Ebioscience	Ter119	TER-119
Invitrogen	CD19	Ebio(ID3)
Invitrogen	CD3	17A2
Invitrogen	FoxP3 (IN)	FJK-16s
Invitrogen	NK1.1	PK136
Invitrogen	TCRβ	H57-597
Biolegend	CD25	PC61-
Biolegend	CD49b	HMα2

### Imaging

For histological analysis by light microscopy, median liver lobes were fixed in 10% NBF for 24 h, dehydrated through a series of graded alcohols and embedded in paraffin blocks. Tissues were cut into 5 μm thick sections using a microtome and stained with Masson’s Trichrome (MT; granulomatous inflammation). For quantification of granulomatous pathology and fibrosis, MT stained liver sections were scanned with a dotslide digital slide scanner (Olympus BX51, VS-ASW FL Software), creating differential Interference Contrast (DIC) images of the whole slide (see **Figure 5A**). Using FIJI imaging analysis software (ImageJ 1.48r) (45) individual liver sections were cropped to obtain solely liver sections. Colour thresholds were set using hue, saturation and brightness (HSB) settings to select either granulomas alone (blue staining) or liver parenchyma (purple staining). HSB thresholds were set using an algorithm developed by Dr. Tim Kendall (Western General Hospital, Edinburgh), enabling the quantification of the number of pixels for the region of interest.

For immunohistochemistry (IHC) analysis by confocal microscopy, pieces of the left liver lobe were placed in optimum cutting temperature formulation (OCT) and stored at -80°C until further processing. Tissues were cut into 20 μm cyrosections, dried overnight at RT, then fixed in ice-cold acetone for 5 min before storage at -20°C. For confocal Immunohistochemistry, cryosections were thawed and rehydrated with 1X PBS before incubation for 1 h at RT with 10% blocking solution: 1% BSA, 10% FCS and 0.005 μg/µl FcR-Block (2.4G2) in PBS-T (Tween-20; 1:1000 in PBS). Samples were incubated overnight at 4°C with fluorescently labelled monoclonal antibodies (all eBioscience) for CD11c (N418; 1:250), Siglec-F (E50-2440; 1:100) TCRβ (H57-597; 1:50), in 2% blocking solution (0.2% BSA, 2% FCS and 0.005 μg/ul FcR-Block in PBS-T). Stained cryosections were washed once with PBS-T followed by 2 washes with PBS, then stained with 1 μg/ml DAPI dissolved in PBS for 15 min at RT. Cryosections were subsequently mounted with coverslips using ProLong Gold antifade reagent (LifeTechnologies) and blinded before analysis. Analysis of confocal photographs was carried out using Volocity imaging software (PerkinElmer). For each photograph, the contrast was enhanced to remove background staining and schistosome eggs were cropped out of individual images to exclude possible autofluorescence during subsequent quantification steps. Snapshots of cropped images were captured separately for each fluorochrome used, and for each snapshot the surface area of positive staining above a fixed intensity was measured. Intensity was set using isotype controls for each fluorochrome labelled.

### RNA Extraction and Sequencing

RNA extraction was performed in line with Peña-Llopis & Brugarolas (46). In brief, liver tissue samples weighing between 15 mg and 40 mg were homogenized in lysis buffer, followed by an acid-phenol chloroform extraction of total RNA. Polyadenylated mRNA was purified from liver total RNA using oligo(dT) Dynabead selection, followed by metal ion hydrolysis fragmentation with the Ambion RNA fragmentation kit. First strand cDNA was synthesized using randomly primed oligos followed by second strand synthesis using DNA polymerase I to produce double-stranded cDNA. This was followed by end repair with T4 and Klenow DNA polymerases and T4 polynucleotide kinase to blunt-end the DNA fragments. A single 3’ adenosine nucleotide was added to the repaired ends using a Klenow fragment (3’→5’ exo-) and dATP to deter concatemerization of templates, limit adapter dimers and increase the efficiency of adapter ligation. Adapters were then ligated (containing primer sites for sequencing). Ligated fragments were run on an agarose gel, size selected for 100-200 bp inserts and the DNA extracted according to the Qiagen gel extraction kit protocol, except for the dissolution of gel slices, which was done at room temperature rather than 50°C. Libraries were then amplified by PCR to add primers for flow cell surface annealing and to increase yield; sample cleanup was performed with AMPure SPRI beads. The libraries were quantified on an Agilent Bioanalyzer and Kapa Illumina SYBR Fast qPCR kit, and sequenced on the Illumina HiSeq 2500 platform as 75 bp paired end reads.

### RNAseq Analysis

mRNA reads were aligned to the mouse reference transcriptome (GRCm38) using Bowtie2 v2.2.1 (47) and quantified using eXpress v1.3.0 (48). Briefly, the mouse cDNA reference was downloaded from the Ensembl Genomes FTP server and indexed prior to mapping using the Bowtie2-build command. Reads were aligned with Bowtie2 using default parameters apart from -X 800, which sets the maximum fragment length for concordant pairs at 800 bp, thus ensuring that reads from all valid fragments are considered; and -a, which reports all alignments. The Bowtie2 alignments were passed directly to eXpress for transcript quantification. As eXpress reports transcript-level counts, a custom perl script was used to aggregate and collapse the reported counts into their corresponding gene-level counts. The results were organized into a tab-delimited matrix with each row representing a gene and each column representing a sample.

Read counts were normalized and differential expression analysis performed using the DESeq2 package (49). Genes with low expression were removed prior to downstream analysis by removing those where the sum of the total read counts in all samples was fewer than 50. Gene names were assigned to ensembl IDs using the biomaRt package (50). Principle component analysis was performed using the DESeq2 package. For the production of volcano plots and heatmaps genes were considered significantly differentially expressed between conditions if they had an adjusted p value (padj) < 0.01 and a log2FoldChange in expression >1. Gene set enrichment analysis for GO terms associated with biological process was performed using the clusterProfiler package (51). Genes with padj < 0.01 (both up- and down-regulated) were selected for gene set enrichment analysis. The statistical significance of enriched terms was adjusted using the Holm method. GO terms with a padj > 0.01 were considered statistically significant. Redundant GO terms were removed using the ‘simplify’ function in clusterProfiler using a cutoff value of 0.5 when selecting by padj.

### Statistical Analysis

Statistical analyses were performed using GraphPad Prism 9 or JMP software. As described in corresponding figure legends, experimental groups were compared using an unpaired t-test, one-way or two-way ANOVA, followed by appropriate *post-hoc* tests. Significance for all statistical tests was shown in figures as P < 0.05 (*), P < 0.01 (**), P < 0.001 (***).

## Results

### Dynamics and Development of Hepatic Pathology

**Figure 1:**
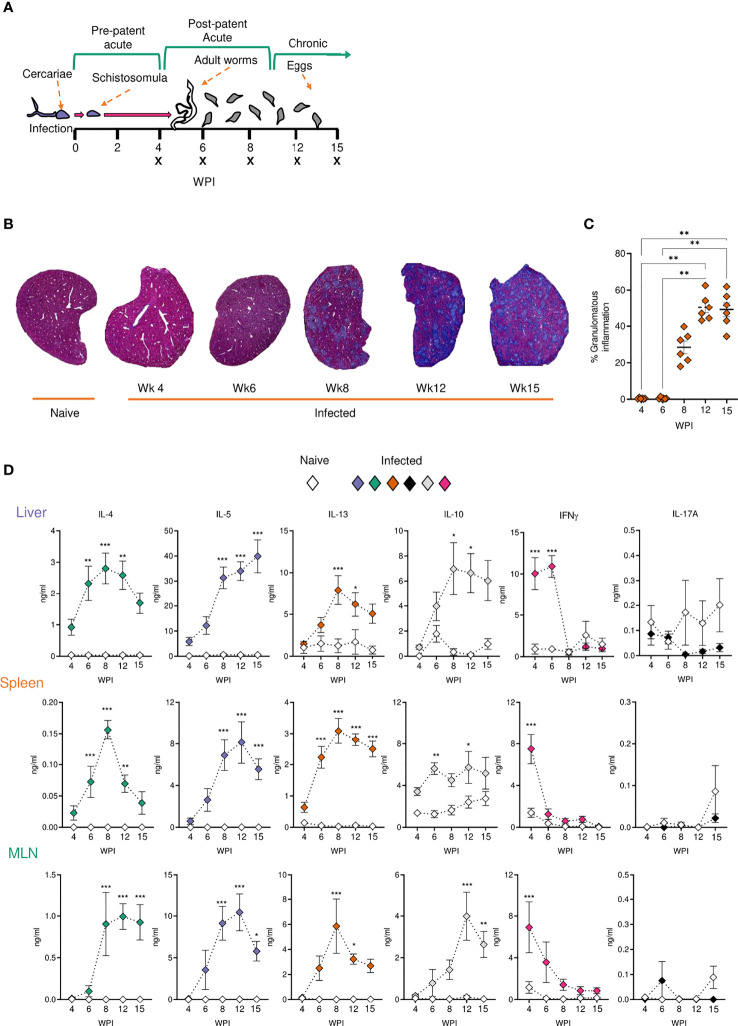
Development of granulomatous pathology and a dominant Type 2 response during *S. mansoni* infection. **(A)** Schematic of infection setup. C57BL/6 mice were infected with 40 *S. mansoni* cercariae with infections lasting, 4, 6, 8, 12 or 15 wks in duration (indicated by X). **(B)** Representative images of liver sections stained with Masson’s Trichrome (MT) at indicated wks, allowing for visualisation of inflammatory cell infiltration and type I collagen deposition. **(C)** The proportion of granulomatous inflammation per tissue section, using an objective algorithm to quantify the number of pixels of granulomatous inflammation in a defined region of interest. **(D)** At specified wks, liver, spleen and MLN cells from naïve and schistosome infected mice were cultured for 72 h with 0.25 µg of schistosome egg antigen (SEA; antigen-specific stimulation). Supernatants were collected and cytokine production (medium alone values subtracted) was assessed by ELISA. Data are from a single experiment **(B, C)** or pooled from 2 **(D)** separate experiments (n=36-10 animals per time point). Significance calculated by one-way **(C)** or two-way ANOVA **(D)**. Data presented as mean +/- SEM. *p < 0.05, **p < 0.01, ***p < 0.001.

Experimental schistosome infections of mice are commonly employed to model the pathological and immunological features of human infections (52, 53). To assess host responses at several distinct stages of schistosomiasis, mice were percutaneously infected with 40 cercariae, and animals taken for analysis after 4, 6, 8, 12 or 15 wks (**Figure 1A**). In brief, these wks correspond to pre-egg production/adult pairing and maturation (wk 4), the initial phase of egg production (wk 6), the peak of post-patent active disease (wk 8), progression into chronic disease (wk 12) and established chronicity (wk 15) (2-4). Egg-driven granulomatous responses generally peak at 8 wks post infection before declining in magnitude, with calcified and fibrotic granulomas accumulating during chronic infection stages, coincident with the immune response against freshly laid eggs being down-regulated (36, 54, 55). Importantly, as egg production is an ongoing, asynchronous process, infected tissues simultaneously present with newly formed, mature and resolving granulomas. To visualise the extent of granulomatous inflammation in the liver, sections from the median lobes of infected mice were stained with Masson’s Trichrome (MT) (**Figure 1B**). Before the start of egg-laying (wk 4), there were no visible signs of damage or eggs in the livers of infected mice, whilst at later infection stages, eggs continued to accumulate within hepatic tissue (**Figure 1A** and **Supplementary Figure 1A**). Granulomatous inflammation became more pronounced at each ensuing stage, with infected livers showing extensive MT staining from wk 8, which accounted for approximately 50% of the total tissue section by wks 12 and 15 (**Figures 1B, C**). Closer assessment of wks 6-8 revealed that granulomatous inflammation in this transition phase started at approximately 45 days post infection, with significantly greater inflammation visualized by day 51 (**Supplementary Figures 1B, C**) and with evidence of enhanced DC, T cell and eosinophil infiltration to hepatic tissue, as assessed by confocal microscopy paired with quantification of CD11c^+^, TCRβ^+^ and Siglec-F^+^ staining (**Supplementary Figure 1D**). Furthermore, mirroring the exaggeration of hepatic pathology during later stages of infection (**Figures 1B, C**), infected mice displayed significantly higher serum levels of SEA-specific antibodies and IgE by wks 12 and 15 (**Supplementary Figures 1E, F**), as well as hepatosplenomegaly from wk 8, as defined by increased spleen and liver weight as a proportion of total body weight (**Supplementary Figure 1G**).

### Tissue-Specific Cytokine Responses During Infection

Next, to define how parasite-specific cytokine responses develop across infection, we focused on three tissue sites that are differentially affected by schistosomiasis: the liver, spleen, and MLNs. More specifically, while the liver represents a principal effector site in which eggs deposit and granulomas form, the spleen and MLNs represent important immune priming sites against parasite Ags from systemic circulation and intestinal sites, respectively.

To evaluate Ag-specific cytokine secretion, isolated liver, spleen and MLN cells were cultured in the presence of SEA (**Figure 1D**). Irrespective of the tissue site in question, immune cells from infection produced very low levels of SEA-specific Th2 associated cytokines (IL-4, IL-5 and IL-13) at wk 4 post infection, with no statistically significant differences relative to naïve mice. In contrast, the Th1 associated cytokine IFNγ peaked at this time-point in infected animals. As infections progressed beyond wk 6 and splenic, MLN and hepatic IFNγ dramatically reduced. All tissues showed increased production of IL-4, IL-5 and IL-13 from wk 6, which either peaked at 8-12 wks post infection or remained elevated into chronic stages. IL-10 responses tended towards an increase during later stages of infection, which was particularly evident in the MLNs and liver. Similar cytokine profiles were seen upon stimulation of isolated cells with anti-CD3 (**Supplementary Figure 2**). This included peak Th2 cytokine potential at approximately 8 wks post infection, and increased IL-10 during chronicity, particularly in the liver and MLNs (**Supplementary Figure 2**). Moreover, while SEA-specific IL-17A production was minimal in all three sites, anti-CD3 stimulated liver cells from infected mice produced significantly increased levels of IL-17A than their naïve counterparts.

### Cellular Responses to Schistosome Infection Vary Across Tissues

**Figure 2:**
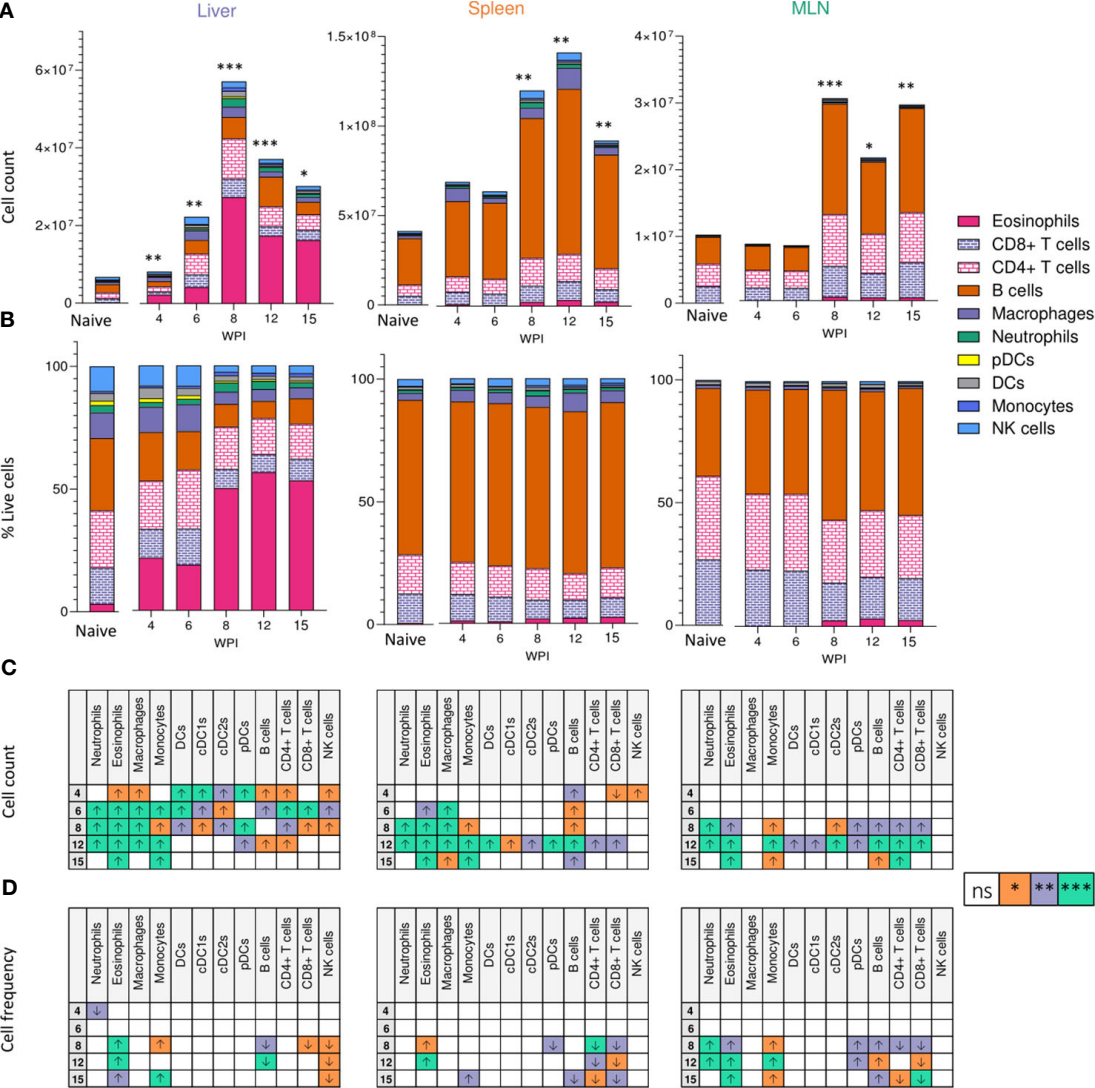
Tissue-specific cellular responses during schistosomiasis. Stacked bar charts showing hepatic splenic and mesenteric **(A)** cell counts and **(B)** cell frequencies (as a proportion of total live cells) at indicated wks of infection when infected with 40 cercariae. For infected mice, data is presented as mean values for each given time point, with averages calculated from two pooled experiments per time point. n=6-8 per timepoint from two pooled experiments. Naïve data is presented as mean values for the entire infection, with averages calculated from two pooled time course experiments. n=30. Significance in **(A)** reflects comparison of total cell counts between naïve and infected mice. Statistics tables showing differences in **(C)** cell counts and **(D)** cell frequencies between naïve and infected mice, for the liver spleen and MLN. Arrows in table **(C, D)** represent the direction of cell frequency change in infected animals in comparison to naïve. Significance calculated by Kruskal-Wallis followed by Dunn’s multiple comparisons test, with comparison between naïve and infected groups. *p < 0.05, **p < 0.01, ***p < 0.001, ns = non-significant (P > 0.05).

We next looked to identify the cell types responding at different sites over the course of infection. From as early as 4 wks post infection, despite the lack of egg production, cellular infiltration to the liver was significantly increased in infected animals compared with naïve (**Figure 2A**). Closer inspection of immune cell numbers (**Figures 2A, C**) and frequencies (**Figures 2B, D**) revealed a dramatic increase in hepatic eosinophils, macrophages, DCs, plasmacytoid DCs (pDCs), B cells, CD4^+^ T cells and NK cells in comparison to naïve mice, but without any significant increase in their frequency. Similarly, while wk 6 saw an increase in overall cell numbers in the liver (aside from pDCs), cellular frequencies remained comparable to naïve controls. However, cell proportions altered significantly by wk 8 of infection, with an increased frequency of hepatic eosinophils, neutrophils, and monocytes, coinciding with a decline in B cell, T cell, NK cell, DC and macrophage proportions. These differences in percentages persisted into later wks of infection. Akin to the earlier observed peak in Th2 cytokine production (**Figure 1D**), liver cell counts peaked at 8 wks post infection before a gradual decline (**Figure 2A**). By 15 wks post infection, eosinophils and monocytes were the only cell types found to be significantly more numerous in infected livers than naïve.

Although there were clear differences in basal immune cell composition between priming (MLN and spleen) and effector (liver) sites (**Figure 2B**), changes in cellularity evoked by schistosome infection were largely comparable in all three tissues. MLN and spleen cell counts remained unchanged at wks 4 and 6 when compared to naïve mice but, similarly to the liver, a significant infiltration in immune cells was observed from wk 8 onwards in these sites that persisted into chronic stages of disease. Eosinophilia was a prominent feature of both MLNs and spleens from the 8 wk time-point onwards, but less dramatically than in the liver. Neutrophilia was also evident within MLN and splenic responses by wks 8 and 12 that, akin to the liver, was no longer significant by 15 wks post infection. Notably, the liver and spleen saw a decrease in B cell frequency at later stages of infection, while increased B cell proportions were observed in the MLNs.

**Figure 3:**
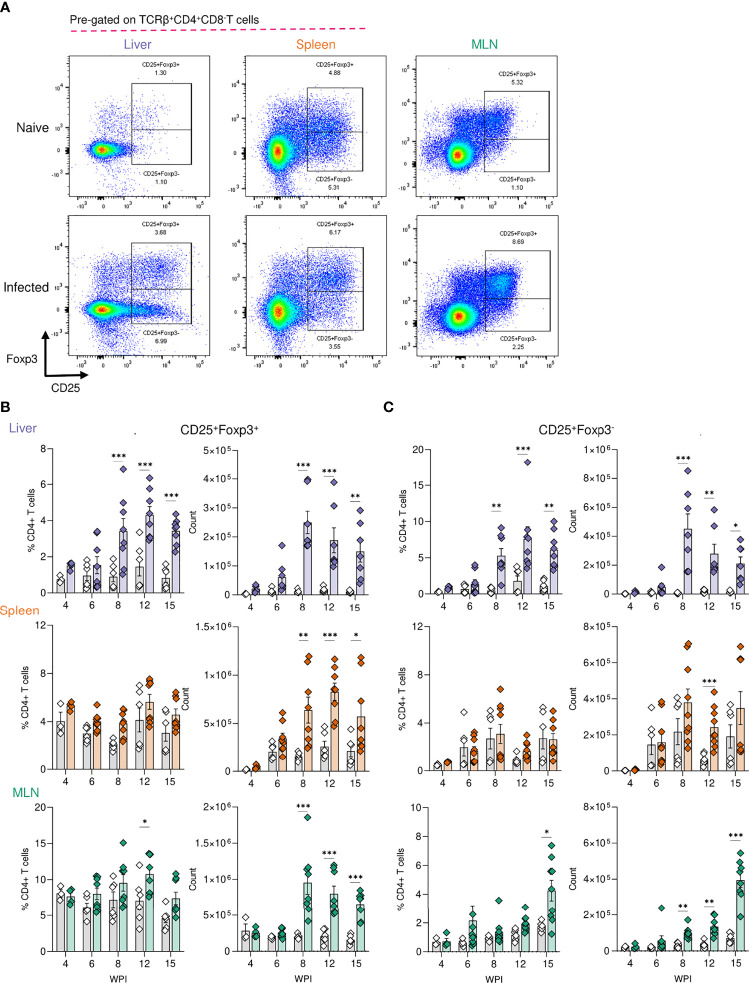
Regulatory T cell dynamics across *S. mansoni* infected tissues. **(A)** Representative flow plots for CD25^+^Foxp3^+^ and CD25^+^Foxp3^-^ gating, pre-gating on live CD45^+^TCRβ^+^CD4^+^CD8^-^ cells. The frequency and total numbers of **(B)** CD25^+^Foxp3^+^ T cells and **(C)** CD25^+^Foxp3^-^ cells in the liver, spleen and MLN of naïve and 40 cercariae infected mice, at indicated timepoints, and with frequency presented as % of total CD4+ T cells. **(B, C)**. Results are mean +/- SEM from two experiments pooled (wks 6-15) or a single experiment (wk4) (n=3-8 mice per group per time-point). Significance calculated by two-way ANOVA. *p < 0.05, **p < 0.01, ***p < 0.001.

Tregs are key immunosuppressive cells that aid in control of immunopathology during *S. mansoni* infection (30, 36, 37, 56), commonly defined by their expression of the transcription factor FoxP3 (57) and/or the activation marker CD25 (IL-2 receptor α) (58) (**Figure 3A**). When looking at relative Treg proportions, we observed significant expansion of CD25^+^Foxp3^+^ Tregs within the liver of infected mice from 8 wk onwards (**Figure 3B**), whilst in the spleen and MLNs, an increase in CD25^+^Foxp3^+^ Treg frequency was observed at wk 12 in the MLNs only (**Figure 3B**). However, reflecting the infection-induced increase in liver, spleen, and MLN cellularity (**Figure 2A**), we observed significant numerical expansion of CD25^+^Foxp3^+^ Tregs by wks 8, 12 and 15 in the liver, spleen and MLNs (**Figure 3B**). Assessment of CD25^+^Foxp3^-^ CD4^+^ T cells, representing activated effector CD4^+^ T cells populations (59), revealed their numerical and proportional expansion in the liver from wk 8 of infection (**Figure 3C**). These Foxp3^-^ populations showed numerical expansion in the MLNs at wks 8, 12 and 15, with significant proportional increases met at wk 15, while the least striking expansion of these effector cells was evident in the spleen (**Figure 3C**).

### Transcriptional Signature of Schistosomiasis Associated Liver Pathology

**Figure 4:**
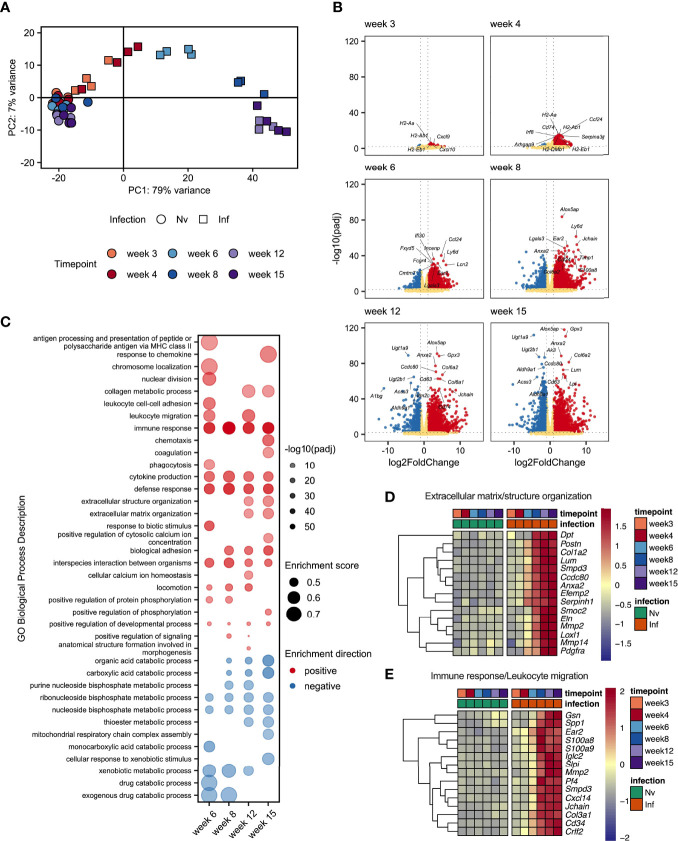
Schistosome egg deposition dramatically alters the liver transcriptome. Livers from schistosome infected mice (Inf) or matched naïve controls (Nv) were harvested at 3, 4, 6, 8, 12 and 15 wks post infection (40 cercariae infection, n=3-4 per timepoint). RNA was isolated from the liver tissue and transcriptionally profiled by RNAseq. **(A)** Principal components analysis of total read counts. Points represent individual replicates. Point shape indicates group, Nv (○) Inf (◻), and colour indicates timepoint. **(B)** Volcano plots of differentially expressed protein-coding genes in Inf vs Nv mice at each time point. Genes were considered significantly differentially expressed in Inf mice when the adjusted p value (padj) was < 0.01 and the log2FoldChange was > 1. Red dots indicate genes significantly up-regulated in Inf mice, blue dots indicate genes significantly down-regulated in Inf mice, yellow dots represent genes not significantly differentially expressed between Inf and Nv mice. **(C)** Gene set enrichment (GSE) analysis for Inf mice from wk 6 to wk 12 using GO Biological Process terms based on genes with a padj < 0.01 vs Nv mice. Points represent the enrichment score for a given GO term at each time point. Size of point indicates the enrichment score. Opacity of point indicates the significance value for that enrichment score expressed as -log10(padj). Point colour indicates whether a given GO term was positively (red) or negatively (blue) enriched. Blank spaces indicate that GO term was not significantly enriched at that timepoint. GO terms were considered significantly enriched if padj < 0.01. Heatmaps representing the mean expression of top 15 most significantly differentially expressed genes identified in specific GO terms: **(D)** “extracellular matrix structure” and “extracellular matrix organisation” and **(E)** “Immune response”, and “Leukocyte migration”. All genes presented by heatmap had a p value < 0.01. Differential expression analysis was performed using DESeq2, GSE analysis was performed using clusterProfiler.

Given the dramatic changes in liver pathology and granulomatous inflammation observed over the course of *S. mansoni* infection, we next sought to obtain a broader, deeper and less biased understanding of the changes occurring in the liver tissue over the course of *S. mansoni* infection. RNA was isolated from the livers of infected mice and matched naïve controls at wks 3, 4, 6, 8, 12, and 15, with transcriptional profiling performed by RNAseq (**Supplementary Excel File 1**). As early as wk 3 post infection we were able to observe distinct transcriptional profiles in infected mice compared to naïve controls (**Figures 4A, B**) including increased expression of chemokines (Cxcl9 and Cxcl10) and genes associated with MHCII expression (*H2-Aa*, *H2-Ab1* and *H2-Eb1*) (**Figure 4B**). From wk 4-6 onward, significant upregulation of transcripts associated with maturation of myeloid cells and B cells (*Irf8*, *Cd74*, *Ly6d* and *Ear2*) was observed. Starting at wk 8 and continuing through wks 12 and 15 we observed collagens (*Col6a1* and *Col6a2*) and other genes associated with the extracellular matrix (ECM) (*Lpl*, *Lum*, *Anxa2*) among the most significantly upregulated differentially expressed genes (DEGs; **Figure 4B**).

To obtain an overview of the processes influenced by infection we performed gene set enrichment analysis (GSE) of gene ontology (GO) terms associated with biological processes using genes differentially expressed at each timepoint (**Figure 4C** and **Supplementary Excel File 2**). Whilst there were too few differentially expressed genes at wk 3 and wk 4 post infection to identify statistically meaningful enrichment of GO terms, we identified a range of terms to be enriched (both positively and negatively) from wk 6 onwards. As expected, we observed strong positive enrichment of terms associated with the immune response, including those related to antigen presentation, immune cell migration and cytokine production. Notably, our GSE analysis identified an number of terms associated with the synthesis of collagen and restructuring of the extracellular matrix at wk 12 and wk 15. Interestingly, we identified strong negative enrichment of a number of GO terms associated with metabolic processes, particularly those associated with the metabolism of nucleotides.

As we had observed substantial hepatic changes by MT staining (**Figure 1B**) we examined genes associated with extracellular matrix remodelling. Selecting the 15 most significant DEGs associated with GO terms relating to tissue remodelling revealed strong upregulation of matrix metalloproteases (MMPs) (*Mmp2* and *Mmp14*); a collagen (*Col1a2*) and ER-resident chaperon associated with collagen biosynthesis (*Serpinh1*); other secreted components of the extracellular matrix (*Dpt*, *Postn*, *Efemp2*, *Smoc2* and *Elna*); a catalytic enzyme required for matrix component crosslinking (*Loxl1*), a matrix binding protein (*Ccdc80*); and receptors associated with the regulation of matrix deposition (*Anxa2* and *Pdgfra*) (**Figure 4D**). In total we identified 76 DEGs associated matrix, including 13 encoding collagens, 5 Adamts (A Disintegrin and Metalloproteinase with Thrombospondin motifs) family members, 8 MMPs, and the transcription factor *Sox9* (**Supplementary Excel File 1**).

Interrogation of the most significantly differentially expressed genes associated with the immune response revealed a diverse array of genes linked to different aspects of immune function. We identified genes associated with immune cell chemotaxis (*Cd34*, *Cxcl14, Ear2* and *Pf4* (encoding CXCL4)); TLR4/RAGE binding S100 family members associated with triggering migration *via* NF-κB signalling (*S100a8* and *S100a9*); an actin regulator associated with macrophage function (*Gsn*); genes associated with interaction with and remodelling of the extracellular matrix (*Col3a1, Mmp2, Spp1*); genes related to B cell function including an alarmin receptor involved in B cell development (*Crlf2*) and components of immunoglobulins (*Iglc2* and *Jchain*); a signal transduction enzyme (*Smpd3*); and an antimicrobial peptide (*Slpi*) (**Figure 4E**).

These data demonstrated a dramatic change in the transcriptional profile of the liver during *S. mansoni* infection with strong enrichment of genes linked to the attraction and activation of leukocytes, and the restructuring of ECM. Additionally, we found significant downregulation of GO terms associated with various metabolic and catabolic processes. Although some of these changes were evident as early as 3 wks post infection, the major changes in gene expression occurred between wk 6 and wk 8, indicating that transcriptional reprogramming of the liver environment is associated with the start of egg production.

### Hepatic Granuloma Composition Over the Course of Infection

**Figure 5:**
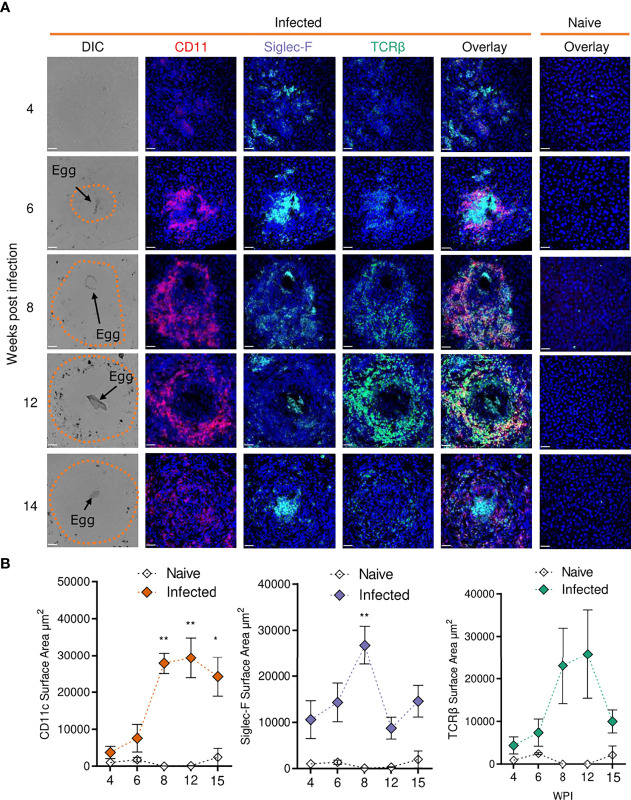
Confocal microscopy analysis reveals distinct alterations in hepatic granuloma composition across infection timeline. At indicated stages of 40 cercarial dose infection, the infiltration and localization of CD11c^+^, Siglec-F^+^ and TCRβ^+^ cells in hepatic granulomas was assessed by IHC. **(A)** Representative confocal microscopy images taken from livers of 5 *S. mansoni* infected mice at each timepoint. Top row showing differential Interference Contrast (DIC) images, with eggs indicated by arrows and dotted lines outlining granuloma periphery. **(B)** Quantification by Image J of positive Siglec-F, CD11c and TCRβ staining. 1 experiment. Significance calculated by Two-way ANOVA. Data presented as mean +/- SEM. *p < 0.05, **p < 0.01. Bars indicate SEM of from 3 sections of naïve hepatic tissue vs 10 granulomas from infected mice.

We next sought to investigate some of the transcriptional changes we had observed at a location level, in hepatic granulomas (**Figure 5**). Schistosome granulomas consist of ECM, fibroblasts and a range of immune cells (predominantly Th2 cells), whose frequency and spatial distribution varies across the course of infection and the site in question (55, 60). To examine the spatial dynamics of granuloma formation and resolution and assess the localisation of key cell types, confocal microscopy was performed on liver sections at each stage of infection (**Figure 5A**).

While no eggs were found in livers from infected mice at wk 4, we observed disorganised clustering of immune cells with sporadic Siglec-F staining throughout that was not present in naïve mice (**Figure 5A**). By wk 6, immune cells began to assemble into more organised, compact, areas that enveloped tissue-trapped eggs and harboured CD11c^+^ and Siglec-F^+^ cells, and a small number of TCRβ^+^ cells, at their core. By wks 8 and 12, CD11c^+^ and Siglec-F^+^ cells now appeared distributed across granulomas, with new infiltration and co-localisation of TCRβ^+^ cells with CD11c^+^ cells. By wk 15, CD11c^+^ and TCRβ^+^ cells reduced in frequency but remained dispersed and colocalised across granulomas, while Siglec-F^+^ cells were again evident within the innermost layer, along with some staining across granulomas. By quantifying the surface area for positive staining around individual eggs, we were able to assess the kinetics of these cell types within granulomas (**Figure 5B**). From wks 6-8, the staining for each marker increased markedly, but was only significant for CD11c and Siglec-F. After wk 8, Siglec-F decreased while CD11c remained consistently elevated until wk 15. TCRβ patterns were somewhat comparable to CD11c, but with a more dramatic reduction by wk 15. These histological data were generally consistent with the hepatic flow cytometry readouts for DCs, eosinophils and T cells (**Figure 2**).

### CD11c Depletion Dramatically Impairs Hepatic Immune Cell Polarisation, Provoking Neutrophilia

**Figure 6:**
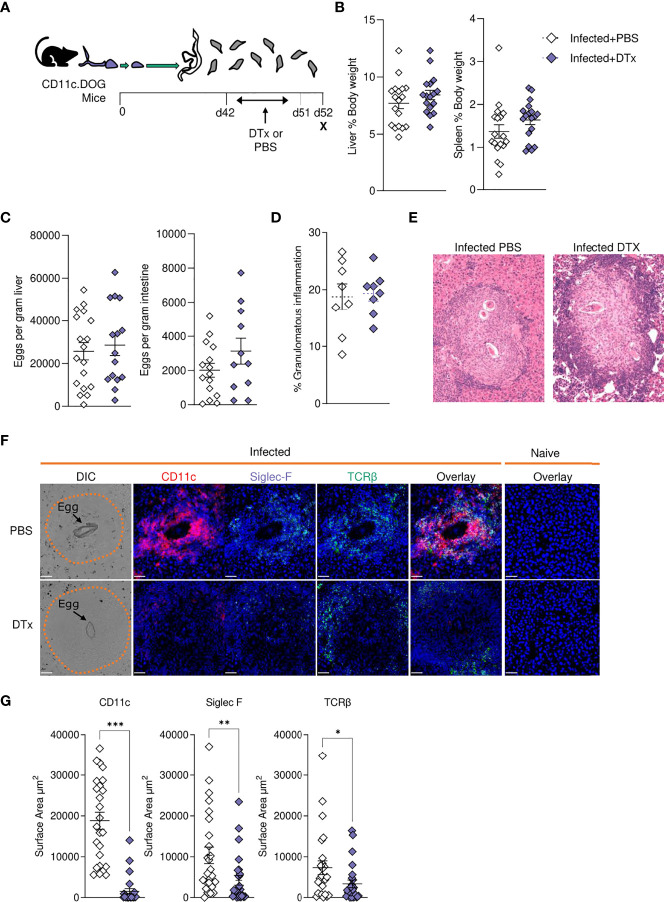
CD11c depletion disrupts granulomatous pathology during *S. mansoni* infection. **(A)** Schematic of infection setup. CD11c.DOG mice were infected with 40 *S. mansoni* cercariae with CD11c^+^ cells depleted *via* Dtx administration on days 42-51, and mice culled at d52. **(B)** Liver and spleen weights for infected mice with data represented as a proportion of total body weight. **(C)** The total number of schistosome eggs per gram of liver or intestinal tissue. **(D)** Quantification of granulomatous inflammation. **(E)** Representative images of hepatic granulomas stained with H&E. **(F)** Representative confocal microscopy granuloma images, with staining for CD11c, Siglec-F and TCRβ. First column showing differential Interference Contrast (DIC) images, with eggs indicated by arrows and dotted lines outlining granuloma periphery. **(G)** Quantification of positive Siglec-F, CD11C and TCRβ staining. Data are from a single experiment **(D–G)** or pooled from 3 **(A–C)** 3 separate experiments. Significance calculated by unpaired T-test. Data presented as mean +/- SEM. *p < 0.05, **p < 0.01, ***p < 0.001.

In previous work, we used targeted CD11c^+^ cell depletion to reveal a crucial role for CD11c^+^ cells in Th2 induction during initial egg producing stages of *S. mansoni* infection (wk 4-6) (24), prior to the dramatic pathological and immunological changes that occur from 6–8-wks post infection (**Figures 1**–**5**). We next sought to explore how CD11c depletion would affect immunopathology and the maintenance of immune responses at later stages of infection using CD11c.DOG mice, in which the CD11c promotor controls expression of the human diphtheria toxin (DTx) receptor (DTR) (40). CD11c^+^ cells were depleted from day 42 to 51 of infection, and host responses evaluated at day 52 (**Figure 6A**). CD11c depletion had no discernible impact on hepatosplenomegaly (**Figure 6B**), egg burden (**Figure 6C**) or the extent of hepatic granulomatous inflammation, as measured by quantification of MT staining (**Figure 6D**). However, closer inspection of hepatic granuloma composition revealed more inflammatory pathology following CD11c depletion (**Figure 6E**). As expected, CD11c^+^ staining was significantly reduced in granulomas from DTx treated mice, indicating effective depletion (**Figures 6F, G**), coincident with a significant decrease in the proportion of TCRβ^+^ and Siglec-F^+^ cells (**Figures 6F, G**).

**Figure 7:**
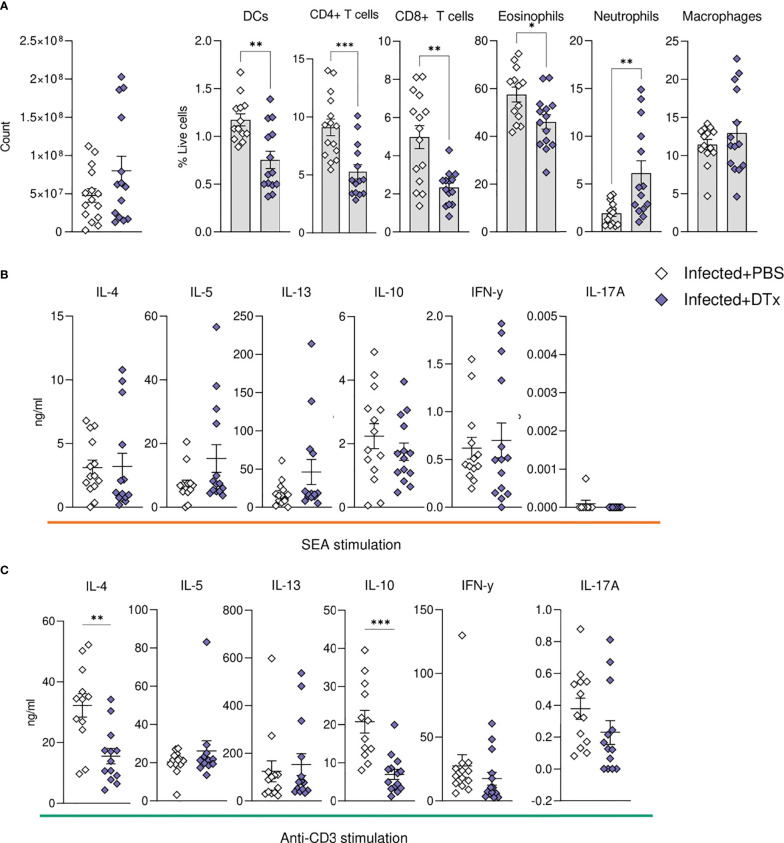
CD11c depletion compromises hepatic cellular dynamics during *S. mansoni* infection. CD11c.DOG mice were infected with 40 S. mansoni cercariae, with CD11c+ cells depleted on d42-51 via Dtx administration, and mice culled on d52. The total number of liver cells **(A)** and the frequency of various immune cells in the liver of Dtx of PBS treated infected mice. Liver cells were cultured for 72 h with **(B)** 0.25 µg of SEA or **(C)** 0.5μg of anti-CD3. Supernatants were collected and cytokine production (medium alone values subtracted) was assessed by ELISA. Data are pooled from 3 separate experiments (n=12-18 animals per time point). Significance calculated by unpaired T-test. Data presented as mean +/- SEM. *p < 0.05, **p < 0.01, ***p < 0.001.

In support of our histological data (**Figures 6F, G**), assessment of liver cell populations by flow cytometry showed that DTx administration significantly depleted CD11c^+^MHC-II^+^ DCs (approximately 50% depletion across experiments), while hepatic macrophage proportions remained intact (**Figure 7A**). These changes were accompanied by a significant reduction in hepatic CD4^+^ T cells, CD8^+^ T cells and eosinophils, alongside a stark increase in hepatic neutrophilia (**Figure 7A**). Next, to explore the impact of CD11c depletion on cytokine dynamics, we evaluated hepatic responses following culture of isolated liver cells with SEA (**Figure 7B**) or anti-CD3 (**Figure 7C**). While CD11c depletion did not significantly alter Ag-specific cytokine production by isolated liver cells in culture, their potential to produce IL-4 and IL-10 in response to anti-CD3 was significantly diminished (**Figure 7C**). Together, these data showed that CD11c depletion significantly influenced the hepatic granulomatous response during schistosome infection, reducing eosinophilia and T cells, while increasing neutrophilia and impairing IL-4 and IL-10 potential, without dramatically affecting Ag-specific cytokine production.

## Discussion

In addition to elevating our basic understanding of immune response development over the course of murine *S. mansoni* infection, in both effector and priming sites, we have revealed a crucial role for CD11c^+^ cells in hepatic granuloma coordination and maintenance in the post-patent phase of schistosome infection. The onset of egg production evoked pronounced pathological (**Figure 1** and **Supplementary Figure 1**), immunological (**Figures 1**–**3** and **Supplementary Figure 2**) and transcriptional host responses (**Figure 4**), with clear immunopathology by the eighth wk of infection (**Figures 1**, **4**, **5** and **Supplementary Figures 1**, **2**). As demonstrated through cytokine analysis of cultured liver, spleen or MLN cells *ex vivo* (**Figure 1** and **Supplementary Figure 2**), infection elicited a distinctive triphasic kinetic, including a Th1-skewed phase prior to egg production, a Th2-dominated response from egg deposition, and a more regulated profile in the chronic stage (**Figure 1** and **Supplementary Figure 2**). Egg deposition drastically remodelled the liver transcriptome, including dramatic enrichment of genes associated with granulocyte recruitment, tissue remodelling and leukocyte activation, and with the most pronounced transcriptional changes starting between wk 6 and 8 of infection (**Figure 4**). Histologically, distinctive hepatic granulomatous inflammation was evident by wk 6 of infection, which subsided by more chronic stages (**Figures 1**, **5**). Importantly, depletion of CD11c^+^ cells between wks 6-8 of infection had a dramatic impact on the hepatic response, including disrupted granuloma formation and cellularity, increased neutrophilia, and impaired cytokine production (**Figures 6**, **7**).

### Time Course Kinetics

We found that the dramatic granulomatous inflammation that develops in the liver from wk 6 of infection (**Figure 1**) became evident over the course of a matter of days, with day 45 representing a distinct tipping point between ‘homeostasis’ and stark pathology (**Supplementary Figure 1**). However, it is important to note that, although we selected representative images for each timepoint, granuloma development is asynchronous, due to differences in timing and location of egg deposition, egg maturity and secretions, and previous exposure to cross-reactive worm Ags (52, 61). To investigate granuloma development in a more synchronous manner, investigations could be made using intravenous injections into the portal vein or lung (52).

Schistosome infection actively modifies T cell responses in order to promote the Th1-Th2 switch, trigger regulatory cell networks, and induce a state of T cell hyperresponsiveness (8, 62, 63). We observed a reduced propensity for isolated cells from livers, spleens or MLNs of more chronically infected mice to produce cytokines in response to Ag-specific (SEA) stimulation (**Figure 1**), in support of previous studies suggesting CD4^+^ T cell exhaustion during later stages of active disease (64, 65). In contrast, upon polyclonal (anti-CD3) stimulation, many cytokines continued to increase in the liver and MLNs beyond wk 12 of infection (**Supplementary Figure 2**). This might suggest that down-modulation of T cell activation during chronic infection is restricted to Ag-specific responses. Alternatively, it is possible that while Ag-specific T cells enter an exhausted state following repeat Ag stimulation (5), a pool of T cells undergo Ag-independent bystander activation towards circulating cytokines and TLR ligands, thus sustaining cytokine secretion across infection (66). Finally, the reduction in T cell activity may reflect the direct activity of schistosome products on T cells or modulation of APC activity (19, 65, 67, 68), which could include promoting the emergence of more tolerogenic APCs (65, 68) or those with a reduced capacity to elicit effector Th2 cell proliferation (69).

In keeping with regulatory responses being more apparent during chronicity (5, 29), we observed evidence for enhanced immunoregulation in later stages of infection (**Figures 1**, **2** and **Supplementary Figure 2**). CD25^+^Foxp3^+^ Tregs were expanded across all tissues at later infection phases, but with the most statistically robust expansion (both proportional and numerical) within the liver (**Figure 3**). These observations support previous studies showing enhanced Treg frequencies in the liver, MLN, spleen and colonic granulomas during infection (30, 36, 37) and suggest the hepatic inflammatory environment to most effectively support Treg activation and recruitment. This may result from the costimulatory environment (63), TGFβ (38), RELMα (70, 71) egg antigens (35, 37), or the higher levels of IL-4 and IL-13 (**Figure 1**), all of which have shown capable of inducing CD25^+^ Tregs from peripheral naïve CD25^-^CD4^+^ T cells (72, 73). Although previous work has assessed Treg development in the spleen, MLN and liver during murine schistosomiasis (30, 36, 37), we are the first to show side by side comparison of tissue specific Treg kinetics over the course of infection. It remains to be addressed whether Breg and CD8^+^ Treg (74) generation mirrors these CD4^+^ Treg dynamics, and whether *S. mansoni* elicited Tregs harbour tissue-specific functions.

We also observed increased IL-10 levels as infection progressed, from isolated and cultured spleen, MLN and liver cells, with this profile being most evident with anti-CD3 stimulation (**Figure 1** and **Supplementary Figure 2**). This may reflect our choice of SEA for Ag-specific *ex vivo* restimulation, given that adult worm-derived molecules may preferentially expand regulatory responses (19, 68). Splenic IL-10 levels were low in comparison to the liver and MLN, which again could reflect the choice of stimulation or the relative abundance of cultured cell types. Furthermore, B cells can be meaningful sources of IL-10 during schistosomiasis (29, 34), quantification of which would require alternative approaches, such as stimulation *via* TLRs and CD40 (75), or the use of IL-10 reporter mice, which constitute an improved tool for visualisation of which cell types contribute to the IL-10 pool (76).

In terms of other cellular responses, CD8^+^ T cells comprised a third of all T cells in the liver (**Figure 2**), with previous literature accrediting them with ‘suppressor’ functions in the downmodulation of egg-driven pathology (77, 78). Eosinophils showed increases across tissues during infection, a likely reflection of elevated levels of IL-5 (**Figure 1** and **Supplementary Figure 2**) or chemokines such as CCL24 (79) (**Figure 4**) or CXCL12 (80). Despite their dominating presence within schistosome infected tissues, the true function of eosinophils in schistosomiasis is not yet known, with eosinophil ablation (81) or IL-5 removal (82) failing to impede granuloma formation or fibrosis, influence hepatocellular damage, or impact Th2 development (83). Analysis of Siglec-F staining of liver sections showed eosinophilia during early stages of granuloma formation within the inner perimeter of circumoval inflammation (**Figure 5**). In previous reports, this localisation has only been shown through morphometric inspection of Giemsa or H&E-stained granulomas (55, 60, 81), and this pattern suggests that eosinophils may play an important role in granuloma formation and protection of parenchymal tissue, and/or in the destruction of the entrapped eggs (84). Indeed, an interesting feature of the hepatic response was that liver cells from infected mice had a greater potential to produce IL-5 as early as wk 4 of infection (**Figure 1** and **Supplementary Figure 2**), which could support the rapid recruitment of eosinophils during granuloma initiation and development from around wk 6. Moreover, we suggest that the hepatic immune infiltration observed at wk 4 (**Figure 2**) may be encouraged by the deposition of worm-derived antigens or regurgitation products within the liver. Indeed, schistosome-derived hemozoin has shown to deposit in large quantities the liver and influence components of host immunity, including alternative activation of macrophages (85). Further interrogation of this pre-patent inflammation would ideally entail comparison between naïve, mixed-sex and single-sex infections.

Transcriptomics is a powerful investigative tool, that offers a high-resolution and unbiased description of host processes at an RNA level. The transcriptional profile of the mammalian host during schistosomiasis has been explored in a range of primate and rodent models (86–88), with more recent work exploring the human transcriptome during active infection (89, 90). However, only a few of these studies have interrogated the dynamics of host gene expression at multiple time points of schistosome infection (88), and no comprehensive overview of how the hepatic transcriptional environment changes across the course of infection yet exists. By defining hepatic transcriptional signatures during pre-patency, our data offers insight into first cellular responders and the mechanisms underlying their recruitment (**Figure 4**). Notably, the IFNγ-inducible chemokines *Cxcl9* and *Cxcl10 (* 91) were among the most upregulated genes at wks 3 and 4 post infection, which is consistent with reports from murine *S. japonicum* infection (86) and suggests Th1-associated chemokines (91) may be associated with initial inflammatory cell recruitment and instigation of granulomatous inflammation. Similarly, expression of IFNγ-driven *Tgtp1* (T cell-specific GTPAse-; **Supplementary Excel File 1**), which is associated with M1 effector functions (92) links with literature showing enhanced frequency of M1-like macrophages during acute schistosomiasis (93, 94) and provides further clarity into the timing of their arrival and/or proliferation.

The transcriptome of the post-patent liver (**Figure 4**) clearly reflected the diverse populations of immune cells populating the tissue (**Figures 2**, **3**), as well as the structural and fibrotic changes the liver undergoes to counteract and/or compensate for egg-induced inflammation (**Figures 1**, **5**). From wk 6, infected livers transitioned into an inflammatory state, as evidenced by enhanced transcripts of acute-phase serum amyloid proteins, SAA1 and SAA2 (**Supplementary Excel File 1**), and moderate upregulation (relative to later wks of infection) of chemokines *Ccl8* and *Ccl24* (**Supplementary Excel File 1**). Interestingly, and in line with our histological analysis for Siglec-F^+^ cells (**Figure 5**), the chemotactic ligand for eosinophils, *Ccl24* [encoding eotaxin-2 (95)], reached peak expression at wk 8 of infection before declining at more chronic phases (**Supplementary Excel File 1**). The list of upregulated genes from wk 8 onwards was dominated by genes associated with tissue restructuring and fibrosis, as well as the recruitment, activation and function of key immune cells, including mast cell proteases, eosinophilic elastases and a marker of macrophage alternative activation, chitinase-like 3 (*Chil3*, encoding Ym1; **Supplementary Excel File 1**), whose function during schistosomiasis has yet to be resolved. Our transcriptomic analysis may assist in the identification of novel cellular and molecular targets for the therapeutic control of schistosomiasis, or treatment of schistosomiasis associated hepatic fibrosis. Notably, the ratio of MMPs to tissue inhibitors of matrix metalloproteinases (TIMPs) **Figure 4**), is a suggested determining factor in the severity of schistosomiasis and may provide clarity on the differences in wound healing response and outcome between *S. japonicum* and *S. mansoni* infections (86, 96). Moreover, in terms of individual genes of interest, *Timp-1, Ccl24*, and *Sox9* all demonstrate sustained upregulation from the point of egg production and are all implicated in the exaggeration of fibrosis (97–99). Finally, the transcriptome of the chronically infected liver showed very interesting metabolic changes, including the downregulation of the cytochrome P450 family (CYPs), amongst other drug metabolising enzymes. Importantly, the downregulation of Cyp2b and Cyp3a (**Supplementary Excel File 1**) may have significant implications for the metabolism and clearance of the anti-schistosome drug praziquantel (100) and thus this could represent a therapeutically fruitful area of follow-up.

### CD11c Depletion

Having generated a high-resolution picture of hepatic schistosomiasis at a transcriptional (**Figure 4**) and cellular (**Figures 2**, **3**, **5**) level, we next sought to identify the importance of CD11c^+^ cells in coordinating the liver response to infection. The development of Th2 immunity is crucial for the control of excessive schistosome associated pathology (8, 10, 32, 38). Notably, mice in Th1 polarised settings (including IL-4 deficient or IL-10/IL-4 double-deficient mice) exhibit rapid weight loss at the onset of egg production, elevated expression of proinflammatory Th1 cytokines and mediators, and high levels of mortality between wks 8-10 of infection (10, 32, 101). The mechanisms underlying the dramatic transition from a mixed, low level Th1/Th2 to a Th2 dominated immune response have been thoroughly dissected, with central involvement of STAT-6 (102), IL-4 (10, 32) and IL-4Rα (9, 103) signalling, and DCs (24). However, there is less understanding of how granulomas and hepatic Th2 responses are maintained and regulated over the course of infection. Herein, we show CD11c depletion during peak development of schistosome-elicited Type 2 inflammation (wks 6-8) compromised hepatic granuloma composition (**Figure 6**). Although CD11c depletion at this stage of infection resulted in impaired liver cell potential to produce IL-4 and IL-10 (**Figure 7C**), it did not significantly impact Ag-specific cytokine responses (**Figure 7B**). This contrasts CD11c depletion at wks 4-6, which we have previously shown significantly reduces hepatic cell Ag-specific IL-4, IL-5 and IL-13 production (24). These data indicate that, while CD11c^+^ DCs may be crucial for the induction and expansion of CD4^+^ T cells in priming stages of infection (24) at later stages effector T cells can competently produce cytokines without their assistance, with other APCs such as B cells or macrophages potentially playing larger roles in effector T cell activation at later phases of infection (104). Alternatively, as hepatic CD11c depletion was incomplete in our experiments (**Figures 6**, **7**), it is possible that residual CD11c^+^ DCs were sufficient to maintain hepatic Th2 effector responses. While Ag-specific liver cell cytokine responses were not significantly affected following CD11c depletion at this timepoint, production of both IL-4 and IL-10 was impaired following stimulation with anti-CD3, implicating CD11c^+^ cells in dictating hepatic CD4^+^ T cell potential to produce these cytokines during schistosomiasis. Taken together, these results suggest that CD11c^+^ APCs play a supportive role in stimulation of T cell cytokine production in the liver effector site. In contrast, CD11c depletion dramatically altered granuloma and hepatic cellularity (**Figures 6**, **7**), in particular significantly reducing eosinophilia and T cells, without reducing overall granulomatous inflammation (**Figure 6D**). Deficits in hepatic recruitment and/or retention of eosinophils and T cells following CD11c depletion may fit with the known ability of DCs to produce a range of chemokines (105–107). In line with this, in a model of schistosome egg induced pulmonary granulomatous inflammation, depletion of known eosinophil (105) and Th2 cell (108) chemokines CCL17 and CCL22, which can be produced by DCs (105–107), led to altered granuloma structure, including a dramatic reduction in eosinophilia (109).

In our experiments, neutrophils, which are normally minor constituents of *S. mansoni* granulomas due to the release of egg-derived chemokine-binding proteins and proteases that inhibit neutrophil function or recruitment (16, 110), significantly increased in the liver following CD11c depletion (**Figure 7**). The fact that schistosome eggs actively secrete neutrophil inhibiting molecules suggests that their presence is undesirable during infection, and their expansion may give rise to more damaging hepatic inflammation, as seen in murine *S. japonicum* infection (111). The cause of neutrophilic expansion is unclear, but this has been reported in other studies using CD11c DTR transgenic mice (112). We predict that the dysregulated granulomas observed in CD11c-depleted mice could have severe pathological consequences in later disease stages, with the absence of intact granuloma barrier leading to increased perfusion of hepatotoxic egg molecules into the tissues.

Together, these data indicate that CD11c^+^ cells play a critical role in recruitment or retention of eosinophils and T cells in the liver from wk 6 of schistosome infection, while other CD11c^-^ cells are generally sufficient for reactivation of cytokine production by hepatic effector T cells. Our results provide a platform for future interrogation of the role of CD11c^+^ cells in more chronic stages of infection, alongside employment of more targeted CD11c depletion approaches (113), to enable a more holistic understanding of the role and importance of CD11c^+^ APC subsets in governing immunopathology in effector sites such as the liver over the course of schistosome infection

In conclusion, this study provides a detailed and comprehensive analysis of immune response development over the course of schistosome infection at a resolution not previously achieved, providing a valuable resource to inform future work aiming to better understand the mechanisms that govern immunity, inflammation and pathology in this important neglected tropical disease.

## Data Availability Statement

The unprocessed fastq files relating to the RNA sequencing data presented in the manuscript are deposited in the European Nucleotide Archive, accession numbers SAMEA2668137 - SAMEA2668182. Processed read counts, and the results from the differential expression and gene set enrichment analyses are available in **Supplementary Material**.

## Ethics Statement

All animal experiments were conducted under licenses granted by the Home Office UK, in accordance with local guidelines and following ethical review by the University of Manchester or the University of Edinburgh, and performed in accordance with the United Kingdom Animals (Scientific Procedures) Act of 1986.

## Author Contributions

ASM and AP-A conceived the study. AP-A, AKM, JB, CO, PC, and SB performed the experiments. AC, SC, AP-A, CO, and ASM analyzed the data. AC and SC wrote the manuscript. AP-A, PC, MB, HS, and ASM read the manuscript and provided critical comments. All authors contributed to the manuscript and approved the submitted version.

## Funding

This work was supported by the MRC (G0701437 and MR/W018748/1) and MCCIR core funding (to ASM), and The Wellcome Trust (098051 to MB). PCC is supported by a Wellcome Trust Sir Henry Dale Fellowship (218550/Z/19/Z) and the MRC Centre for Medical Mycology at the University of Exeter (MR/N006364/2).

## Conflict of Interest

Author RL was employed by company 360biolabs.

The remaining authors declare that the research was conducted in the absence of any commercial or financial relationships that could be construed as a potential conflict of interest.

## Publisher’s Note

All claims expressed in this article are solely those of the authors and do not necessarily represent those of their affiliated organizations, or those of the publisher, the editors and the reviewers. Any product that may be evaluated in this article, or claim that may be made by its manufacturer, is not guaranteed or endorsed by the publisher.

## Supplementary Material

Supplementary Excel File 1 Differential expression analysis from RNAseq on the liver during S. mansoni infection **(A)** Read counts for each identified transcript in individual samples **(B)** Sample meta data **(C)** Results from differential expression analysis comparing naive and infected mice irrespective of timepoint **(D)** Results from differential expression analysis comparing naive and infected mice at week 3 post infection **(E)** Results from differential expression analysis comparing naive and infected mice at week 4 post infection **(F)** Results from differential expression analysis comparing naive and infected mice at week 6 post infection **(G)** Results from differential expression analysis comparing naive and infected mice at week 8 post infection **(H)** Results from differentia expression analysis comparing naive and infected mice at week 12 post infection **(I)** Results from differential expression analysis comparing naive and infected mice at week 15 post infection.

Supplementary Excel File 2 Results from gene set enrichment analysis, using Gene Ontology (gseGO) terms associated with biological processes, for genes identified as differentially expressed in the livers of mice infected with S. mansoni compared to naive mice. **(A)** gseGO results at week 4 post infection **(B)** gseGO results at week 6 post infection **(C)** gseGO results at week 8 post infection **(D)** gseGO results at week 12 post infection **(E)** gseGO results at week 15 post infection

Supplementary Figure 1 Development of hepatic pathology and antibody responses during *S. mansoni* infection. **(A)** The total number of schistosome eggs per gram of liver or intestinal tissue. **(B)** Visualisation of hepatic granulomas at 36, 39, 42, 45, 48 and 51 days post infection (DPI). Representative images of liver sections stained with Masson’s Trichrome (MT) **(C)** Quantification of granulomatous inflammation. **(D)** Quantification of positive CD11C, TCRβ and Siglec-F staining in liver sections. **(E)** SEA-specific IgG, IgG1, IgG2c and IgG3 titres in the serum of naïve and infected mice, presented as endpoint serum dilutions. **(F)** Serum IgE titres, presented as concentration. **(G)** Liver and spleen weights for infected mice with data represented as a proportion of total body weight. Data are from a single experiment **(B–F)** or pooled from 2 **(G)** or 3 separate experiments **(A)**, with 3-18 mice per timepoint, per infection group. Significance calculated by one-way or two-way ANOVA. Data presented as mean +/- SEM. *p < 0.05, **p < 0.01, ***p < 0.001.

Supplementary Figure 2 Development of a dominant Type 2 response across tissues during *S. mansoni* infection. Liver, spleen and MLN cells from naïve or infected mice were cultured for 72 h in the presence of 0.5ug anti-CD3. Supernatants were collected and cytokine production (medium alone values subtracted) was assessed by ELISA. Data are pooled from two separate experiments. Significance calculated by two-way ANOVA. Data presented as mean+/-SEM, *p < 0.05, **p < 0.01, ***p < 0.001.

## Abbreviations

Ag, Antigen; APCs, Antigen presenting cells; DCs, Dendritic cells; IL, Interleukin; MT, Masson’s Trichrome; MLN, Mesenteric lymph nodes; Bregs, Regulatory B cells; Tregs, Regulatory T cells; SEA, Schistosome soluble egg Ag; Th, T helper; WPI, Wks post infection.
