# Corrigendum: Dynamics of host immune response development during *Schistosoma mansoni* infection

**DOI:** 10.3389/fimmu.2023.1229665

**Published:** 2023-08-10

**Authors:** Alice H. Costain, Alexander T. Phythian-Adams, Stefano A. P. Colombo, Angela K. Marley, Christian Owusu, Peter C. Cook, Sheila L. Brown, Lauren M. Webb, Rachel J. Lundie, Jessica G. Borger, Hermelijn H. Smits, Matthew Berriman, Andrew S. MacDonald

**Affiliations:** ^1^ Lydia Becker Institute of Immunology and Inflammation, University of Manchester, Manchester, United Kingdom; ^2^ Department of Parasitology, Leiden University Medical Center, Leiden, Netherlands; ^3^ Wellcome Sanger Institute, Wellcome Genome Campus, Hinxton, United Kingdom; ^4^ Medical Research Council Centre for Medical Mycology, University of Exeter, Exeter, United Kingdom; ^5^ Department of Immunology, University of Washington, Seattle, WA, United States; ^6^ 360biolabs, Melbourne, VIC, Australia; ^7^ The Walter and Eliza Hall Institute, VIC, Australia; ^8^ Wellcome Centre for Integrative Parasitology, University of Glasgow, Glasgow, United Kingdom

**Keywords:** schistosomiasis, dendritic cells, pathology, chronic infection, transcriptomic (RNA-seq)

In the published article, there was an error in the author list, and author Jessica G. Borger and their affiliation were erroneously excluded. The corrected author list and affiliation appear above.

The new ‘Author contributions’ section appears below.

## Author contributions

ASM and AP-A conceived the study. AP-A, AKM, JB, CO, PC, and SB performed the experiments. AC, SC, AP-A, CO, and ASM analyzed the data. AC and SC wrote the manuscript. AP-A, PC, MB, HS, and ASM read the manuscript and provided critical comments. All authors contributed to the manuscript and approved the submitted version.

In the published article, there was an error in **Figure 2** and **Figure 6** as published. In **Figure 2**, the splenic cell count (**Figure 2C**) and frequency tables (**Figure 2D**) were erroneously mixed up. In **Figure 6F**, the overlay image for the granuloma of CD11c depleted/Dtx injected mice was incorrect. The corrected **Figure 2** and **Figure 6** including the captions appear below.

**Figure 2:**
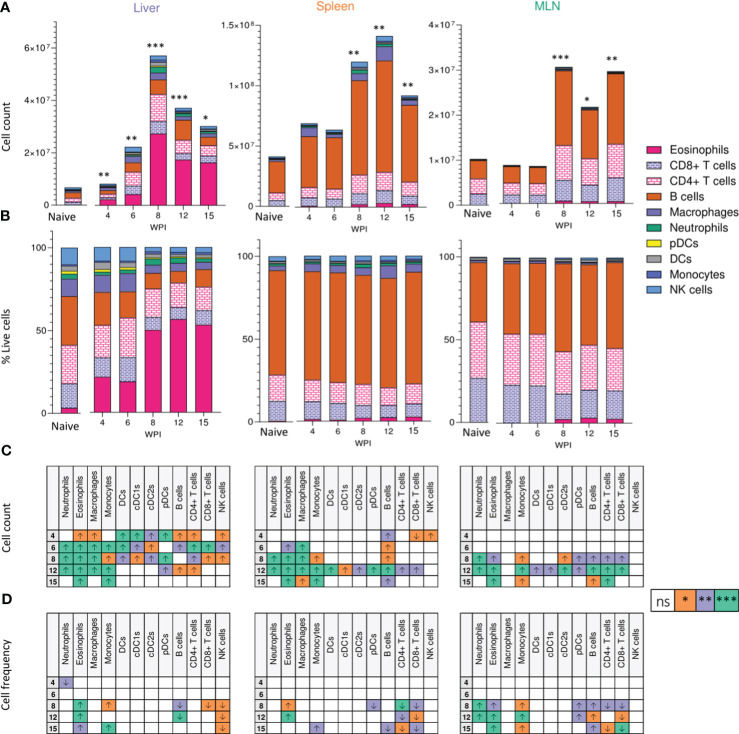
Tissue-specific cellular responses during schistosomiasis. Stacked bar charts showing hepatic splenic and mesenteric **(A)** cell counts and **(B)** cell frequencies (as a proportion of total live cells) at indicated wks of infection when infected with 40 cercariae. For infected mice, data is presented as mean values for each given time point, with averages calculated from two pooled experiments per time point. n=6-8 per timepoint from two pooled experiments. Naïve data is presented as mean values for the entire infection, with averages calculated from two pooled time course experiments. n=30. Significance in **(A)** reflects comparison of total cell counts between naïve and infected mice. Statistics tables showing differences in **(C)** cell counts and **(D)** cell frequencies between naïve and infected mice, for the liver spleen and MLN. Arrows in table **(C, D)** represent the direction of cell frequency change in infected animals in comparison to naïve. Significance calculated by Kruskal-Wallis followed by Dunn’s multiple comparisons test, with comparison between naïve and infected groups. *p < 0.05, **p < 0.01, ***p < 0.001, ns = non-significant (P > 0.05).

**Figure 6:**
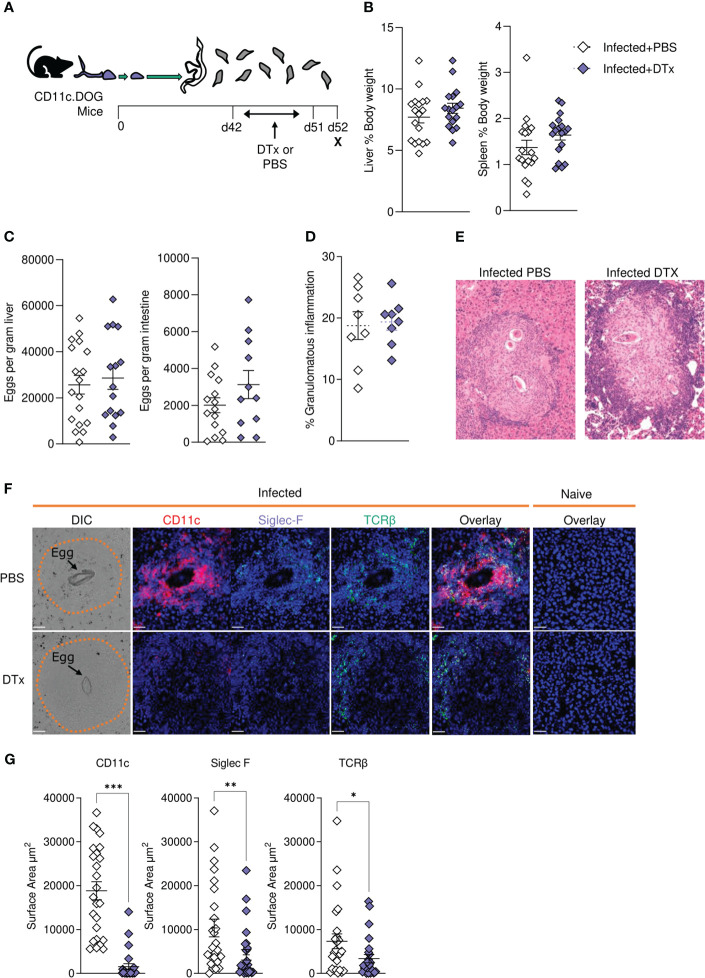
CD11c depletion disrupts granulomatous pathology during *S. mansoni* infection. **(A)** Schematic of infection setup. CD11c.DOG mice were infected with 40 *S. mansoni* cercariae with CD11c^+^ cells depleted via Dtx administration on days 42-51, and mice culled at d52. **(B)** Liver and spleen weights for infected mice with data represented as a proportion of total body weight. **(C)** The total number of schistosome eggs per gram of liver or intestinal tissue. **(D)** Quantification of granulomatous inflammation. **(E)** Representative images of hepatic granulomas stained with H&E. **(F)** Representative confocal microscopy granuloma images, with staining for CD11c, Siglec-F and TCRβ. First column showing differential Interference Contrast (DIC) images, with eggs indicated by arrows and dotted lines outlining granuloma periphery. **(G)** Quantification of positive Siglec-F, CD11C and TCRβ staining. Data are from a single experiment **(D-G)** or pooled from 3 **(A–C)** 3 separate experiments. Significance calculated by unpaired T-test. Data presented as mean +/- SEM. *p < 0.05, **p < 0.01, ***p < 0.001.

In the published article, there was an error. One sentence relating to **Figure 2** was incorrect, due to a mistake in the figure content; detailed above.

A correction has been made to Results, Cellular Responses to Schistosome Infection Vary Across Tissues, Paragraph 2. This sentence previously stated:

“Notably, the liver showed a significant decrease in B cell frequency from wk 8-15 of infection, while increased B cell proportions were observed in the spleen and MLNs.”

The corrected sentence appears below:

“Notably, the liver and spleen saw a decrease in B cell frequency at later stages of infection, while increased B cell proportions were observed in the MLNs”

The authors apologize for these errors and state that these do not change the scientific conclusions of the article in any way. The original article has been updated.

