# Supplementary figures and images for: Dynamics of host immune response development during Schistosoma mansoni infection
Source: Front Immunol. 2022 Jul 8;13:906338. doi: 10.3389/fimmu.2022.906338 (PMC9362740; doi:10.3389/fimmu.2022.906338)

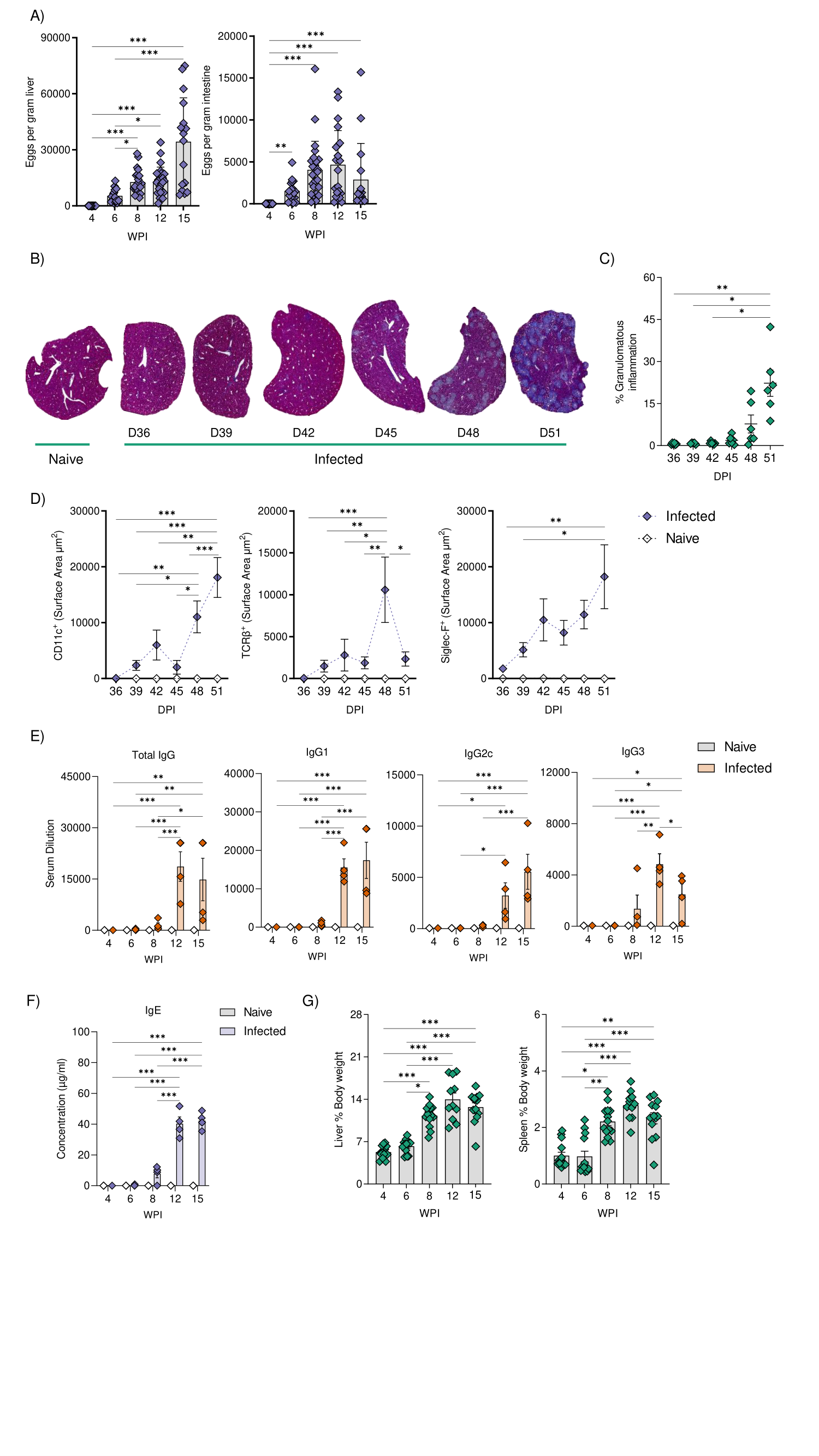

Supplement: Supplementary Figure 1 — Development of hepatic pathology and antibody responses during S. mansoni infection. (A) The total number of schistosome eggs per gram of liver or intestinal tissue. (B) Visualisation of hepatic granulomas at 36, 39, 42, 45, 48 and 51 days post infection (DPI). Representative images of liver sections stained with Masson’s Trichrome (MT) (C) Quantification of granulomatous inflammation. (D) Quantification of positive CD11C, TCRβ and Siglec-F staining in liver sections. (E) SEA-specific IgG, IgG1, IgG2c and IgG3 titres in the serum of naïve and infected mice, presented as endpoint serum dilutions. (F) Serum IgE titres, presented as concentration. (G) Liver and spleen weights for infected mice with data represented as a proportion of total body weight. Data are from a single experiment (B–F) or pooled from 2 (G) or 3 separate experiments (A), with 3-18 mice per timepoint, per infection group. Significance calculated by one-way or two-way ANOVA. Data presented as mean +/- SEM. *p < 0.05, **p < 0.01, ***p < 0.001. [file Image_1.tiff]

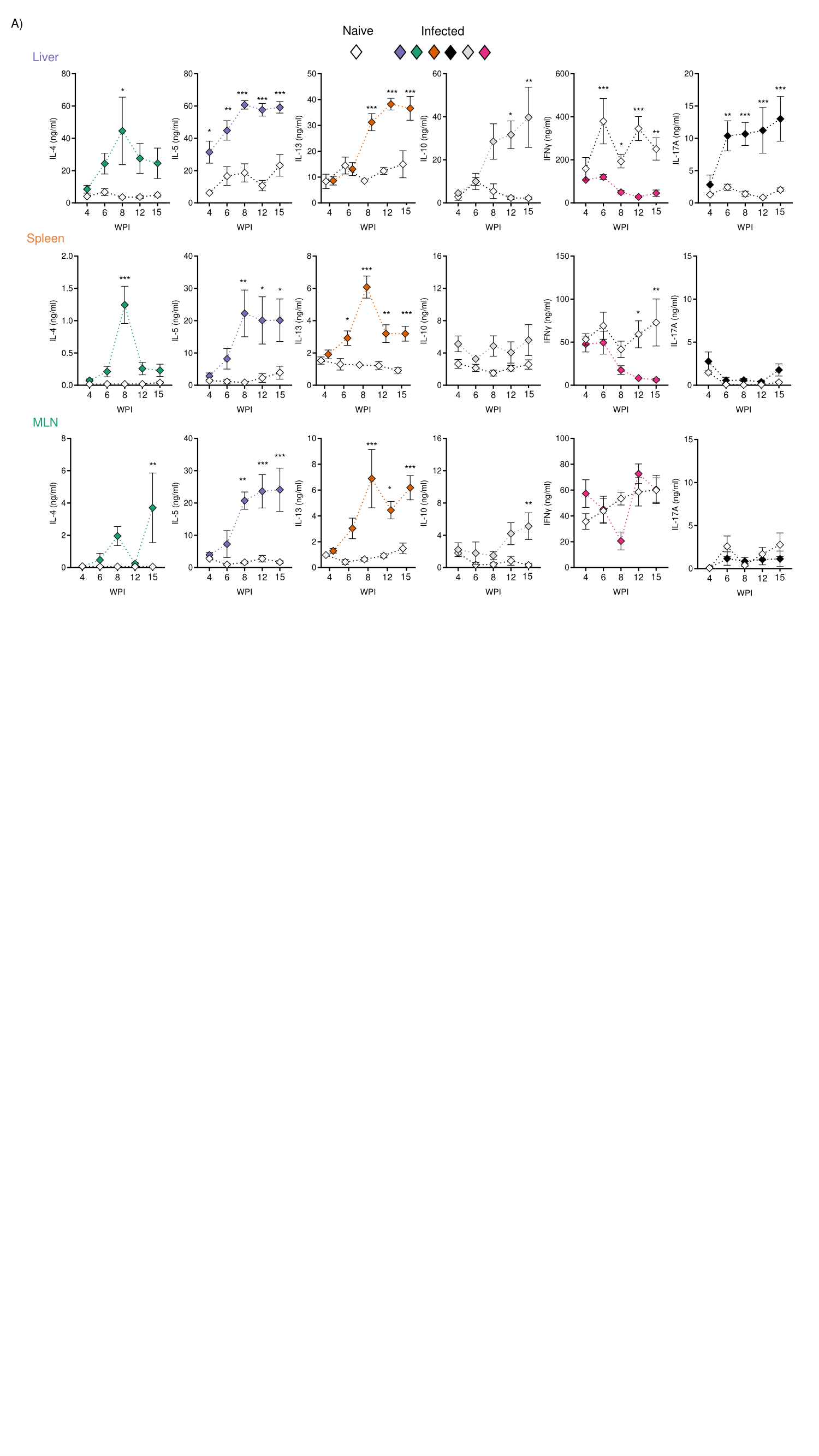

Supplement: Supplementary Figure 2 — Development of a dominant Type 2 response across tissues during S. mansoni infection. Liver, spleen and MLN cells from naïve or infected mice were cultured for 72 h in the presence of 0.5ug anti-CD3. Supernatants were collected and cytokine production (medium alone values subtracted) was assessed by ELISA. Data are pooled from two separate experiments. Significance calculated by two-way ANOVA. Data presented as mean+/-SEM, *p < 0.05, **p < 0.01, ***p < 0.001. [file Image_2.tiff]
